# Privacy and information security risks in a technology platform for home-based chronic disease rehabilitation and education

**DOI:** 10.1186/1472-6947-13-85

**Published:** 2013-08-09

**Authors:** Eva Henriksen, Tatjana M Burkow, Elin Johnsen, Lars K Vognild

**Affiliations:** 1 Norwegian Centre for Integrated Care and Telemedicine, University Hospital of North Norway, Tromsø, Norway; 2 Norut, Northern Research Institute, Tromsø, Norway

**Keywords:** Privacy, Confidentiality, Information security, Risk assessment, Pulmonary rehabilitation, Diabetes self-management education, Video conference, Tele-homecare

## Abstract

**Background:**

Privacy and information security are important for all healthcare services, including home-based services. We have designed and implemented a prototype technology platform for providing home-based healthcare services. It supports a personal electronic health diary and enables secure and reliable communication and interaction with peers and healthcare personnel. The platform runs on a small computer with a dedicated remote control. It is connected to the patient’s TV and to a broadband Internet. The platform has been tested with home-based rehabilitation and education programs for chronic obstructive pulmonary disease and diabetes. As part of our work, a risk assessment of privacy and security aspects has been performed, to reveal actual risks and to ensure adequate information security in this technical platform.

**Methods:**

Risk assessment was performed in an iterative manner during the development process. Thus, security solutions have been incorporated into the design from an early stage instead of being included as an add-on to a nearly completed system. We have adapted existing risk management methods to our own environment, thus creating our own method. Our method conforms to ISO’s standard for information security risk management.

**Results:**

A total of approximately 50 threats and possible unwanted incidents were identified and analysed. Among the threats to the four information security aspects: confidentiality, integrity, availability, and quality; confidentiality threats were identified as most serious, with one threat given an unacceptable level of High risk. This is because health-related personal information is regarded as sensitive. Availability threats were analysed as low risk, as the aim of the home programmes is to provide education and rehabilitation services; not for use in acute situations or for continuous health monitoring.

**Conclusions:**

Most of the identified threats are applicable for healthcare services intended for patients or citizens in their own homes. Confidentiality risks in home are different from in a more controlled environment such as a hospital; and electronic equipment located in private homes and communicating via Internet, is more exposed to unauthorised access. By implementing the proposed measures, it has been possible to design a home-based service which ensures the necessary level of information security and privacy.

## Background

The increasing prevalence of chronic conditions has become a major concern to healthcare systems throughout the world. Healthcare provision faces a shift in emphasis from the management of acute illnesses to the provision of long-term services for people with chronic conditions [1,2]. In this shift, the home environment may have an important role in chronic disease management; for secondary prevention and follow-up, improved self-management skills and quality of life, stabilisation of the chronic disease, prevention of exacerbations, and less hospitalisation.

We have designed and implemented a prototype technology platform for providing home-based healthcare services. It supports a personal electronic health diary and enables secure and reliable communication and interaction with peers and healthcare personnel. The platform runs on a small computer (the Residential Patient Device, RPD) with a dedicated remote control. It is connected to the patient’s TV and to a broadband Internet connection. A web camera and a microphone are used during videoconferencing. By making the technical platform appear as a “smart TV” solution, we also aimed at reaching people less familiar with computers. Our technical platform, including its security measures, has been used in real settings in several trials [3,4].

Privacy and security are important for all healthcare services, including home-based services. As part of our work, a risk assessment of privacy and information security aspects was performed. The motivation for the risk assessment was to reveal actual information security risks and to ensure that the security of the technical platform was adequate. The risk assessment was first performed early in the design process; it was revisited and updated later in the development process. In this way, information security was embedded into the design, in line with the concept of “Privacy by design” [5].

This paper describes the risk assessment and its findings, and the security measures which were implemented. We also discuss some general privacy concerns relating to home-based systems in healthcare.

### The services and the technical platform

The two services subject to risk assessment were two home programmes: a comprehensive pulmonary rehabilitation programme and an education programme for diabetes self-management, both for virtual online groups. The aim of the home programmes is improved access to education and rehabilitation. The home programmes are based on conventional programmes, respectively: 1) a group-based pulmonary rehabilitation programme which encompasses education, exercising, and psychosocial support over several weeks, and 2) a two-day group-based course in diabetes self-management, with a telephone service for individual diabetes consultation.

Both home programmes had weekly group education sessions and individual consultation sessions. In addition, the pulmonary rehabilitation programme had weekly group exercise sessions. An individual consultation with a physiotherapist, pulmonary nurse, or diabetes nurse was introduced as an opportunity for addressing potential personal health matters which they did not want to discuss in the presence of peers. An exercise video (rehabilitation only) and educational videos were also available for the participants.

The patients had a personal electronic health diary for daily use, with a health-related multiple-choice questionnaire, manual input of pulse oximetry values from a self-administered pulse oximeter (Nonin Medical, Inc), and input of blood glucose values from a self-administered OneTouch Ultra Blood Glucose Meter (LifeScan, Inc.) connected through a wireless Bluetooth adapter.

The electronic health diary stores the entered information. The health diary is intended both to support patient self-management and to provide supplementary information for the weekly individual consultation. Each patient explicitly made one week of the health diary data available to the healthcare personnel ahead of each consultation, and the encrypted data were transferred over the Internet from home to a server at the hospital. The healthcare personnel could then access the patient’s health diary before the individual weekly consultation.

The RPD has components for the following functionality: multiparty videoconferencing, storage and visualisation of health diary information, storage and playback of videos, storage and presentation of textual information, wireless incorporation of data from the LifeScan blood glucose meter, and data encryption and transfer of information from home to the hospital information system. The multiparty videoconference component was used for both educational and exercise group sessions and for the individual consultations. The RPD performed no actions or manipulation on the data, except for storage, archival, and communication. Therefore, the RPD is not considered to be a medical device, according to directives 93/42/EEC [6] and 2007/47/EC [7], and based on the EC guidance document MEDDEV 2.1/6 [8].

More information on the platform and services can be found in [3,4,9].

### Legal baseline and security requirements

Requirements concerning the electronic communication of personal information are imposed by national legislation. In European countries these are based on the EU directive on processing of personal data [10]. In the US, similar requirements are based on the Health Insurance Portability and Accountability Act of 1996 (HIPAA) [11]. Under both international and Norwegian legislation, all health-related information concerning an identifiable person is considered *sensitive* (Personal Data Act §2) [12]. No one else than the health professionals who have a treatment relationship to the person should be able to access this person’s health information, unless the person has given his or her consent. In Norway, strong emphasis is put on privacy considerations, also in the healthcare sector.

Most chronically ill patients need, at least to some extent, to have their next-of-kin involved in their care. These individuals are often considered a valuable resource in the treatment and follow-up of the patient. In terms of Norwegian legislation, it is up to the patient to decide whether their next-of-kin should be informed about the details of their condition [13]. If that happens, there is a corresponding exemption from the commitment to professional confidentiality for the healthcare workers [14].

Information security is usually defined as including the three main aspects *confidentiality*, *integrity*, and *availability*[12,15,16]. In the Norwegian healthcare legislation [17], *quality* is included as a fourth aspect of information security. Quality can be seen as overlapping with integrity, and these aspects are often merged in analyses of security threats and risks. However, for applications involving images, video, and audio, it is often useful to manage these two aspects separately. Quality may also be a relevant security aspect in the analysis of applications where usability, ease of use, and user interface are important. The following is a brief definition of the four security aspects:

• **Confidentiality** is the property that information is not made available for or disclosed to unauthorised persons, entities, or processes.

• **Integrity** relates to the trustworthiness of the information. It is the property that data have not been deliberately tampered with, nor accidentally changed.

• **Availability** is the property that information is accessible and usable upon demand by an authorised entity.

• **Quality** refers to the information being correct and not misleading.

Because of the sensitivity of personal health information, confidentiality is particularly important within the healthcare sector. Confidentiality requirements originate from the professional secrecy and non-disclosure undertaking required of all healthcare workers – all the way back to Hippocrates 2400 years ago. The Hippocratic Oath states: “*What I may see or hear in the course of the treatment or even outside of the treatment in regard to the life of men*, *which on no account one must spread abroad*, *I will keep to myself*, *holding such things shameful to be spoken about*[18].”

Privacy is another side of confidentiality. Article 1 of the EU Directive on processing of personal data [10] specifies the “right to privacy with respect to processing of personal data”. A rule of thumb could be to define privacy as the *right* of the client and confidentiality the *duty* of the service provider [19].

## Methods

Security risk analysis is a basic requirement of ISO 27002 [15], internationally recognised as the generic information security standard. In our case, risk assessment is performed with respect to the information security aspects confidentiality, integrity, availability, and quality.

There are two basic types of risk analysis methods: qualitative and quantitative. Quantitative methods use mathematical and statistical tools to represent risk, e.g. by including a calculation of the expected loss of value to an asset. In qualitative risk analysis, risk is analysed by using linguistic variables (adjectives) rather than using mathematics [20]. Qualitative assessment techniques base the risk assessment on anecdotal or knowledge-driven factors [21]. Our risk assessment approach is qualitative. We regard this to be the best approach when assessing new technology being developed for a new model of service delivery. In our case, the final design of technology and service was still to be decided, and there was not much relevant quantitative information available. A qualitative approach opened for discussions of risk aspects in groups involving relevant stakeholders. In these circumstances it is more appropriate to express likelihood and consequence in terms of “adjectives”, instead of unfounded numbers for percentage and money. In addition, methods based on qualitative measures may be more suitable for today’s complex environment of information systems [20].

There are several methods and guidelines for how to conduct risk analysis; they all include the central tasks of

– identifying threats and possible unwanted incidents

– analysing impacts and likelihood of the identified threats

– evaluating risks with respect to acceptance criteria

There seem to be a lack of relevant literature which present risk assessment of information security in healthcare settings, especially within home-based e-health services. Standards for risk assessment in general, as well as textbooks describing ways to perform risk assessment, are available. Organisations often adapt existing risk management methods to their own environment and culture, thus creating their own method [22]. Our method has evolved through several risk assessments [23-25], and it conforms to ISO’s standards for risk management, ISO 31000 [26] and ISO 27005 [27].

The standards set out the risk analysis process in five main steps:

1. **Context establishment**: describe the subject of the analysis, i.e. the system to be analysed and its environment.

2. **Risk identification**: identify which unwanted incidents might possibly happen, i.e. identify threats and vulnerabilities.

3. **Risk analysis**: consider the consequences of the threats and the likelihood that these consequences may occur, i.e. impact and probability analysis.

4. **Risk evaluation**: relate the resulting risk level to the risk acceptance criteria, i.e. decide which risks might be acceptable or not.

5. **Risk treatment**: identify and assess risk treatment options.

Steps 2, 3, and 4 are collectively referred to as “Risk assessment”. In our method, these steps are often performed in a series of risk assessment meetings. Meeting participants are persons with the necessary knowledge about the analysed system. The process is led by a risk assessment expert.

By doing risk assessment in an iterative manner, it becomes an important part of the design and development process. We performed the first risk assessment at an early stage in the design phase, and repeated it as a review and update later on in the development process. In this way, security requirements have been identified, and security solutions were incorporated into the design from the early development phase instead of being included as an add-on to a nearly completed system.

The following sections describe our use of the risk assessment method, step by step.

### Step 1: Context establishment

A high-level description of the system and service and the environment for its use, is necessary in order to perform a risk assessment at an architectural level [21]. In addition to a written description, which is included in the risk assessment report, this is often performed as a walk-through of the system and service, presented at the first risk assessment meeting.

It is also necessary to identify the assets to be protected. In most health-related projects, the patients’ life and health is the main asset. However, in this case the services in question have hardly any potential to harm the patient’s health. But there are other assets to protect, such as the patients’ right to privacy; the hardware and software of the system itself; sensitive information in the system; other healthcare systems and their information, which could be affected from this system; and trust in, and the reputation of, the services and the service provider.

As a part of establishing the context, the risk acceptance criteria are stated during this first phase of the risk assessment. The acceptance criteria are related to the security requirements. It is not possible to completely avoid any risk – to have a risk level of zero. But it is difficult to define that small level of risk which might be acceptable, especially at an early stage of the service and technology development.

It is, however, important to have a common understanding of the acceptance criteria. The following criteria were discussed for our risk assessment:

• A patient should not die or have a reduction in health as a result of using these services.

No such risk would be accepted in these types of services, as for conventional rehabilitation and education programmes.

• Unauthorised persons should not be able to acquire a patient’s health information (confidentiality breach). 

An “unauthorised person” is anyone without a treatment role towards the patient, so this includes family members and relatives. However, for patients with a chronic disease, family members are often considered resource persons in the treatment and follow-up care. In our case, it may be difficult for patients to conceal that they have a chronic disease, especially for COPD patients who are receiving long-term oxygen therapy.

To understand the type and amount of information that might be disclosed is vital for a decision on the acceptability of the risk. In addition to being a legal question, it may also be an individual and subjective question, as to which information should be kept secure. Users may want information to remain private even if it does not concern a matter which is legally defined as “sensitive” information.

• A patient’s health information should not be modified or deleted by unauthorised persons or as a result of software or hardware errors (integrity breach).

By “modify” we mean to insert wrong information, or to alter or remove existing information. As a worst case, this might cause that a patient receive a wrong medical advice or a wrong treatment, but in most cases the only result is destroyed data.

• Stored information should not be permanently lost (availability breach).

In the context of our study, there would be no serious consequences for the patient if data registered in this system were deleted, but it would indicate that the system is not trustworthy. The confidence in and reputation of this system and the services would diminish with an increasing number of such incidents.

• Access to the system and/or its information should not be rendered impossible for those who are entitled to such access (availability breach).

There would be no serious consequence for the patient or the healthcare worker if the service or information were not accessible, but trust in, and the reputation of, the system and the services would diminish on the frequency of unavailability.

At this first step of the risk assessment process, definitions of levels for consequence, likelihood and risk were presented and discussed. In the absence of statistical information or other quantitative information from an operational system, we describe the different levels using qualitative values, sometimes referred to as “linguistic variables” or adjectives [22]. It can be difficult to define the values before threats are identified, and the definitions usually need to be reviewed and updated when they are in use during step 3 of the process. Definitions used in our risk assessment are presented in Table 1.

**Table 1:** **Definitions of values for consequence, likelihood**, **and risk level used in the risk assessment**

***Consequence:***	
**Small**	For the hospital or the service: No violation of law; offence that does not lead to reaction; or negligible financial loss which can be recovered; or small reduction of reputation in the short run. For the patient: No impact on health; or negligible financial loss which can be recovered; or small reduction of reputation in the short run.
**Moderate**	For the hospital or the service: Offence, less serious violation of law which results in a warning or a reprimand; or financial loss which can be recovered; or reduction of reputation that may influence trust and respect. For the patient: No direct impact on health or a minor temporary impact; or financial loss which can be recovered; or some loss of reputation caused by revelation of less sensitive or offensive health information.
**Severe**	For the hospital or the service: Violation of law which results in minor penalty or fine; or a large financial loss which cannot be recovered; or serious loss of reputation that will affect trust and respect for a long time. For the patient: Reduced health; or some financial loss which cannot be recovered; or serious loss of reputation caused by revealing of sensitive and offending information.
**Catastrophic**	For the hospital or the service: Serious violation of law which results in a penalty or fine; or considerable financial loss which cannot be recovered; or serious loss of reputation which is devastating for trust and respect. For the patient: Death or permanent damage of health; or considerable financial loss which cannot be recovered; or serious loss of reputation which permanently affects life, health, and finances.
***Likelihood:***	
**Low**	Rare, occurs less frequently than every 10th year, or less than 10 % of the times the system/service is used. Detailed knowledge about the system is needed; or special equipment is needed; or it can only be performed deliberately and with the help of internal personnel.
**Medium**	May happen, occurs not more than once a year, or between 10 % and 30 % of the times the system/service is used. Normal knowledge about the system is sufficient; or normally available equipment can be used; or it can be performed deliberately.
**High**	Fairly often, occurs several times a year, or between 30 % and 50 % of the times the system/service is used. Can be done with minor knowledge about the system; or without any additional equipment being used; or it can occur because of wrong or careless usage.
**Very high**	Very often, occurs several times a month or more frequent than 50 % of the times the system/service is used. Can be done without any knowledge about the system; or without any additional equipment being used; or it can occur because of wrong or careless usage.
***Risk level:***	
**Low**	Acceptable risk. The service can be used with the identified threats, but the threats must be observed to detect changes that could increase the risk level.
**Medium**	Possibly an acceptable risk for this particular service, but each threat must be considered separately and the development of the risk must be monitored on a regular basis, with an assessment of whether remedial measures should be implemented.
**High**	Unacceptable risk. Cannot start using the service before risk reducing measures have been implemented.

### Step 2: Threat identification

The second step of the risk assessment process, the identification of possible threats and unwanted incidents, was performed as a structured brainstorming between the project members. The brainstorming process was “structured” in the way that a walkthrough of the intended services was performed, using predefined keywords and attributes. Keywords related to the security aspects confidentiality, integrity, quality, and availability, and to attributes such as “internal” and “external” (threats), and “deliberate” and “accidental” (actions). The identified threats and the discussion were summarised in a threat table.

The production of a threat table is a documentation technique which we use throughout the risk assessment process. Brainstorming, with the use of a table to document the threats, corresponds to the technique used in the risk analysis method HazOP [28,29]. We have, however, found it convenient to use some other columns in our threat table. These are:

1. unique identifier of threat (threat number),

2. textual description of threat or unwanted incidence,

3. consequence value,

4. likelihood value,

5. risk value (as a product of consequence and likelihood),

6. any other comments from the brainstorming (including ideas for risk treatment).

During the brainstorming, all possible threats were written into the threat table (column 2), together with any relevant comments (column 6), including those related to consequence and likelihood. Subsequently, the threat table was refined, by grouping related threats, and putting threats into a relevant sequence. At this stage, each threat was given a unique identifier (column 1). Values for consequence, likelihood, and risk were added in later steps of the process.

The threats identified in our risk assessment are listed in Table 2 which corresponds to the first two columns of our threat table.

**Table 2:** List of threats identified in the risk assessment

**ID**	**Threats/unwanted incidents**
*Locally– at the patient’s home*	
c1	Unauthorised persons can view/read personal (sensitive) health information because the user has forgotten to switch off (or “log out” from) the RPD.
c3	Unauthorised persons can view/read personal (sensitive) health information because the PIN code (or password or another authentication mechanism) is available/known – e.g. too weak/simple (a general problem)
c4	Unauthorised persons can view/read personal (sensitive) health information because the RPD with stored information is stolen, then restarted and accessed without authorisation.
c5	Video conference (VC) to participant at home (individual sessions): Unauthorised persons present in the patient’s home, outside camera view, may happen to hear personal information given to **this patient** by health personnel (e.g. instructions/education regarding his/her own disease) Remember: Unauthorised persons are persons (including family members and visitors) with whom the patient does not want to share that information.
c6	Group education via VC (all patients in their own home): Unauthorised persons in a patient’s home, outside camera view, can see and hear **other** patients/participants without their knowledge.
c7	The RPD is compromised because of software weaknesses, making it possible for unauthorised persons to see/log ongoing activity.
c8	Wireless data transfer from sensor to RPD can be intercepted by others.
i1	Unauthorised persons (e.g. grandchildren who play with the sensor) can *by accident* (i.e. unintentionally) insert false values if the system is not fail safe. That is, measures taken from other persons than the registered user are entered.
i3	Unauthorised persons (e.g. other family members or visitors) can *deliberately* insert fake values.
i4	The patient him-/herself can *by mistake* modify inserted values or insert erroneous values (e.g. it is easy to type in wrong O_2_ values).
i7	The patient him/herself can *deliberately* insert fake values or modify inserted values.
i8	Data in the RPD is corrupted - e.g. wrong clock time from a sensor may follow the sensor value and cause existing data to be overwritten.
i9	SW/HW-weaknesses in the RPD that can be exploited (e.g. by malware) in such a way that the information is being damaged or modified.
i10	The RPD is stolen and software, keys or configuration are being exploited for unauthorised communication.
i12	The RPD is being compromised because of SW weaknesses and becomes a relay for attacking healthcare systems, e.g. by sending messages containing executable payload.
i14	Unauthorised persons can remotely configure the RPD, install/update software, etc., thus making the system behave differently than specified.
a1	The service is unavailable for both the patient and the health personnel because the RPD has been stolen.
a2	Data from the RPD cannot be retrieved locally by the patient (SW or HW errors, e.g. disk crash).
a3	Data from the RPD cannot be sent to the health personnel (SW or HW errors).
a4	The RPD is damaged (crushed, fire, dropped to the floor etc.) so that data cannot be retrieved or inserted.
a5	Shutdown because of electricity power failure in the patient’s home.
a6	The patient forgets his PIN-code (or other authentication method) so that data cannot be retrieved from the RPD at home. (Information sent is available at the central server.)
a7	PKI certificates expire. If this happens, it is not possible to send data with valid signatures or to encrypt correctly for the specified recipient.
a8	SW/HW weaknesses in the RPD that can be exploited (e.g. by malware) in such a way that stored information is destroyed/deleted or access is blocked (e.g. Denial of Service attack, DoS)
a9	Patients will not use the system: “Too high-tech”. Fear of surveillance. Feeling of lack of control. Afraid of damaging the system. Think it is difficult to use.
a10	Patients will not use the service because too many errors occur, too often. E.g. in the case of an alert function, error which leads to triggering of the alert.
*During data transfer*	
c9	Unauthorised persons obtain access to personal (sensitive) information during transfer: measurement values from sensors, textual information from patient at home
c10	Unauthorised persons obtain access to personal (sensitive) information being transferred in the two-way video conference, both audio (what is said) and video (see patients in their homes).
i15	Unauthorised persons can modify or delete personal health information during transfer.
i18	Errors during transfer lead to duplication of messages.
a11	Unauthorised persons can delete personal health information during transfer so that it does not reach the intended recipient.
a13	Low network quality (QoS): the quality of the connection is so low that the remote education and exercising is useless.
a14	DoS attack (on the network or a network component) so that the information does not reach the intended recipient.
a15	Low network quality (QoS): data is not transferred, is lost during transfer, or is delayed.
a16	Information corrupted or lost during transfer (caused by errors), i.e. cannot be used by the intended recipient.
*Data in the central server/database, in the health institution*	
c11	Unauthorised persons obtain access to personal health information (in server/database) in the health institution. The server contains information about all patients/participants. If unauthorised persons obtain access, information about several patients can be seen at a time, not just that concerning a single patient.
i21	Information stored on the central server is deliberately manipulated (modified, deleted) by unauthorised persons.
i22	Information stored on the central server is manipulated (modified, deleted) by mistake (e.g. wrong usage)
a17	Permanent loss of data from central server (because of SW errors or HW failures), data are lost or destroyed
a18	Data on the central server are unavailable for a short or a longer time period (e.g. electricity power failure)
*Quality of video communication*	
q1	The video quality from the patient’s home is inadequate (e.g. because of limited bandwidth, camera type, use of camera, placement of camera, lighting, etc.) for the healthcare workers to be able to instruct the patients. They do not see clearly enough what the patient is doing (exercise, use of medical equipment)
q2	Unacceptable audio quality, e.g. echo, jitter, drop-out. The healthcare workers can hear their own echo in the sound from the participants. The patients at home can hear an echo if the healthcare workers do not use an extra microphone

### Step 3: Impact and probability analysis

In step 3 of the process, the identified threats and unwanted incidents were analysed. For each possible threat we asked the project participants to evaluate the impact or consequence and the likelihood that the threat or incident would occur. The qualitative values for consequence and likelihood (as defined in Table 1) were inserted in the threat table, columns 3 and 4, for each identified threat.

It is particularly difficult to set a value for likelihood when the system is at an early stage of development; it is difficult to identify vulnerabilities before the system is built and tested. When deciding on a value for likelihood one could ask relevant questions, for instance related to the ease and motivation for the threats. In our case, the following questions were useful:

– Knowledge, acquaintance: Must an attacker know the configuration of the system? Does he or she need to know a password or PIN? Or is it sufficient to turn on the TV or the computer? Is the system “always on”?

– Remedies: Does an attacker need special equipment, e.g. a smartcard or a code-calculator? Does he or she need access to special software, or would access to normally available software and hardware be sufficient?

– Mistakes: How easy is it to make mistakes? (Mistakes can be caused by insufficient user education or training, or by a poor user interface.)

– Motivation: Would any third party be interested in viewing the information? Would anyone have an interest in deliberately modifying the information?

### Step 4: Risk evaluation

**Figure 1:**
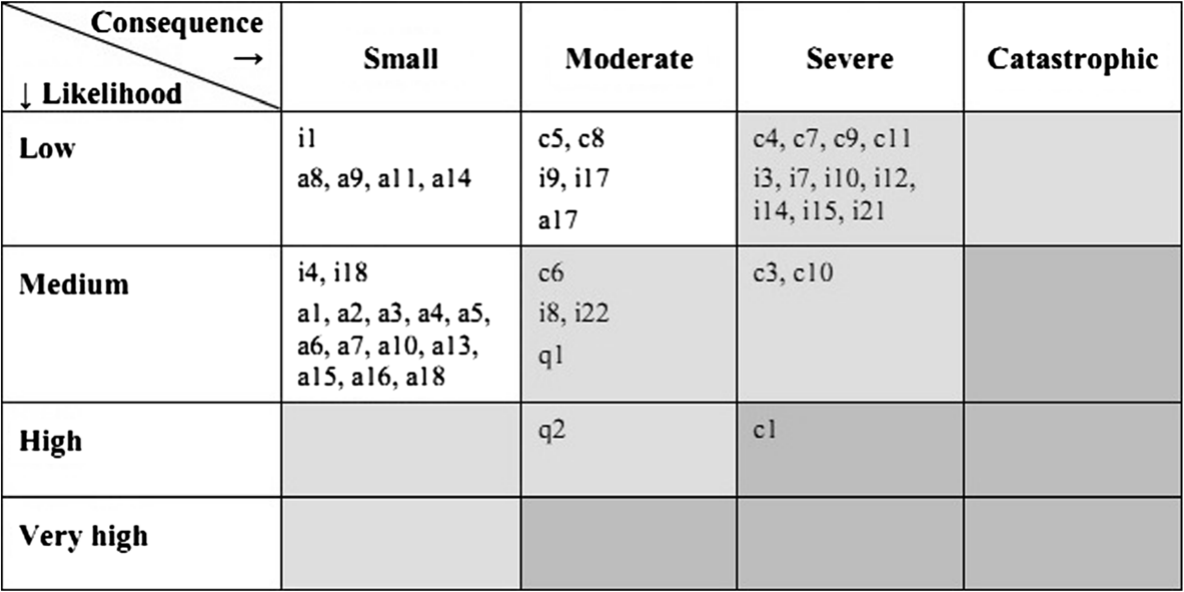
Risk matrix showing risk level for identified threats.

The *risk* of a threat is defined as the product of consequence and likelihood for that threat. This is illustratively represented by a two-dimensional matrix (like Figure 1).

In step 4 of the process, the unique ID of each identified threat was written into the corresponding cell of the matrix. The result of our risk assessment is shown in the risk matrix in Figure 1. The shading of the cells in the matrix indicates the three risk levels defined in Table 1. The risk value of each threat, indicated by the position in the matrix, was also inserted into column 5 of the threat table.

### Step 5: Risk treatment

For all threats with a non-acceptable risk level, risk-reducing treatment was proposed and discussed (step 5 of the risk assessment process). The responsibility for follow-up of the proposed measures is outside the scope of the risk assessment. That responsibility belongs to the project management.

There are basically four different approaches to managing a risk [27].

• **Risk retention** – accept the risk in accordance with the organisation’s security policy. This applies to those risks that are deemed low enough to be acceptable. It is worth remembering that accepting the risk does not mean that the unwanted incident indicated by the threat is acceptable.

• **Risk reduction** – reduce the risk to an acceptable level. Since risk is a product of likelihood and consequence, this implies reduction of the likelihood, the consequence, or both. It is most difficult to reduce the consequence of a threat; the focus should therefore first of all be on reduction of likelihood.

• **Risk avoidance** – not be exposed to the risk, do not do the things that could lead to the risk.

• **Risk transfer** – transfer the risk to a third party (e.g. to an insurance company)

Risk reduction measures should be considered and evaluated with reference to the cost-benefit for the service. Some measures might reduce the risk level for several threats at the same time. Cheap and easy-to-implement measures that are likely to reduce the risk of even an acceptable threat should be implemented as a matter of course.

## Results

### Identified threats

A total of approximately 50 threats and potential unwanted incidents were identified and analysed. Each threat was given a unique identifier whereby the initial letter shows the security category the threat belongs to: c = confidentiality; i = integrity; q = quality; a = availability. – The relevant threats identified are listed in Table 2 (a few threats which were considered irrelevant have been excluded from this summary). Threats to health information can occur in the patient’s home, at the remote server, or during transfer between the system in the patient’s home and the server. The intermediate headings in the table indicate a grouping according to subsystems of the analysed system in question and its context.

### Analysis and treatment of risk

The identified threats were given values for likelihood and consequence. The unique ID of each threat was written into the corresponding cell of the risk matrix, as shown in Figure 1. The risk level indicated by the matrix is analysed, and appropriate treatment of the risk is proposed.

The risk matrix shows that none of the threats was regarded as having a *catastrophic consequence*. For these particular services, this would seem to be a reasonable conclusion, as the aim of the home programmes is to provide education and rehabilitation services; it is not for use in acute situations or for continuous health monitoring. The matrix also shows that none of the threats was considered to have a *very high likelihood*, according to our definitions in Table 1.

The risk matrix indicates that *confidentiality* threats have been identified as the most serious threats. This is because all health-related personal information is defined to be sensitive information in the legal regulations [10,12]. It is also worth noting that all the *availability* threats were identified as *low* risk. This reflects the fact that the services are not intended to be used for real-time monitoring or for acute situations. The health diary acts as supplementary information for the weekly individual consultations. The worst consequence of these threats is probably that the system will lose credibility and the users will fail to trust it, and will stop using the service if there are too many such problems.

The only threat that scored an unacceptable *High* risk level, **c1**, is related to confidentiality. It concerns the case that a third party, e.g. a visitor in the patient’s home, by chance could access personal health information on the TV screen if the system is left switched on and logged-in. The *High* risk is a result of the severe consequence of confidentiality breaches, and the high likelihood of this particular threat.

A technical solution to threat **c1** is to implement a timeout mechanism to be activated after a specified period without interaction between the user and the system. The user has to be authenticated again when reactivating the service. The length of the timeout must be considered carefully; there is a trade-off between security and usability: Too short a timeout will be an annoyance to the user, who has to log in repeatedly, while a too long timeout has limited value.

The rest of the threats discussed here are threats with *medium* risk. These threats may be acceptable according to the risk level definitions in Table 1. It is, however, important to monitor the development of these threats, as their risk may rise to an unacceptable high level in case of increased likelihood. For many of them, it would be appropriate to implement risk-reducing measures.

In addition to c1, there are other confidentiality threats to health information in the patient’s home:

Unauthorised persons, e.g. visitors, in a patient’s home can see and hear other persons participating in the video conference (**c6**). This can happen without the knowledge of the other participants; they will only see persons within the camera’s coverage area. On the other hand, it can be argued that participating in centre-based training courses also increases the likelihood that other persons will know about your chronic disease, since it is possible that family or next-of-kin also participate in some of these courses.

The user has to be authenticated when logging in to the RPD. If a PIN is introduced for logging on to the system, it is quite likely that the patient will write the code on a paper, and thus make it possible for others to access the system (**c3**).

For most of the threats involving the patients in their homes there are also non-technical measures for risk reduction. It is important to give information to the users, make them aware of the risks and explain the reason for restrictions imposed by technical solutions and routines for use. Such education to the users is relevant for threats **c1**, **c3** and **c6**.

Threat **c4** concerns the security of the information stored in the RPD in case the dedicated computer is stolen. The information stored consists of both the values from sensor measurements and manual input from the patient, as well as the education videos made by the healthcare workers. In the design of the system it was decided that medical data stored in the RPD should be encrypted, which gives a very low likelihood for this threat to occur. The education videos were not encrypted; they do not contain information that is sensitive in terms of any legislation.

Unauthorised persons might exploit potential SW weaknesses to hack into the RPD and reveal health information (**c7**). The likelihood of this is greater than zero because this is a prototype system; on the other hand, the opportunities to access the system have been greatly reduced by setting it up as a dedicated PC, with access only via dedicated ports and protocols.

Confidentiality threats also arise during information transfer and when the information is stored in the hospital’s server.

Sensitive information is transferred in both directions over the Internet. A security requirement which originates from the legislation is that sensitive data transmitted over an insecure network, i.e. a network over which the organisation does not have full control, should be encrypted during transfer. So textual data and sensor values are encrypted during transfer, and it is therefore less likely that these data will be revealed (**c9**). We cannot disregard the risk that someone is able to wiretap audio and video during video conferencing sessions (**c10**). This risk was reduced by using Virtual Private Network (VPN) to achieve private encrypted video conferencing over the Internet.

Data residing in the hospital’s server are covered by the same security as the rest of the hospital’s systems, and we consider the likelihood of unauthorised access to these data to be low (**c11**).

Most of the threats related to *integrity* and *availability* are considered to have less consequence for the patient, as long as similar incidents are not repeated over a long period. This is because decisions for the patients concerned are not based on single measurements. On the other hand, if there were systematic incidents over time (such as threats **i3** and **i7** which could concern deliberate registration of wrong values for a long time), this could perhaps lead to more severe consequences for the patient’s health. This is also the case for the integrity of the health information during transfer or while it resides in the hospital server (threats **i15**, **i21**). Taking into account the security measures at the hospital, the likelihood is *low* that the information stored on the server will be deliberately manipulated. However, the data on the server could also be modified by mistake (**i22**), for instance if the user interface is not good enough.

Threats **i10**, **i12** and **i14** all relate to the possibility of obtaining control over the RPD and its content and using it for illegitimate purposes, either by stealing it (**i10**) or by exploiting SW weaknesses to attack the system over the Internet (**i12**). If the dedicated computer can be reached via the network, it might be reconfigured and thus made available for processes other than those initially intended (**i14**). All these are threats with severe consequence, but we regard their likelihood to be very low.

Threat **i8** concerns the possibility that data in the RPD are corrupted because of SW weaknesses or HW problems. If the information still appears to be credible, this might, in the worst case, cause incorrect advice given to the patient; if not, it is just unavailable information as in threat **a16**. The consequence is considered to be moderate. The information from the electronic health diary acts as a supplement to the individual consultation where the patient and the health professional discuss health issues. The information is readable and can therefore be verified by the patient. No automated decision is made based on the information from the diary.

Two threats to *quality* were also identified in the risk assessment, **q1** concerning the video quality and **q2** concerning the audio quality. The quality may be diminished if the bandwidth is too low or the equipment is too poor. It might result in blurred pictures, or delayed video. If, for instance, there is a delay in the video signals, this might be misinterpreted by the physiotherapist as the patient not keeping up with the speed. The audio quality is usually considered more important than the video quality. The most annoying problem would be echoing of the sound, a participant receiving his/her own voice back through the TV set. Training health workers and patients in the use of the system will help to reduce the risk of threats such as **q1** and **q2**.

## Discussion

A risk assessment process is continuous and iterative, and it applies to different phases of the system development [21]. Our risk assessment was performed following this approach. Thus, security aspects were incorporated into the design from an early stage instead of being included as an add-on to a nearly completed system. This is also referred to as “Privacy by design”, information security is embedded into the design; it is proactive instead of reactive, preventive instead of remedial [5].

### Principle results

Of the approximately 50 threats and potential unwanted incidents that were identified and analysed, none were regarded as having *catastrophic* consequence, and only one threat was identified as having an unacceptable *High* risk level. Threats to *confidentiality* were deemed most serious. This is because all health-related personal information is regarded as sensitive. Most of the threats to integrity and quality were analysed to have *Medium* risk, while threats to availability were regarded *Low* risk.

Some security measures were planned already in the design of the system, and these were taken into account as prerequisites in the risk assessment. Additional measures were added as a result of the risk assessment. In summary, the following security measures were included in the design and implementation of the technical platform [3,4,9], reducing the risk accordingly:

– Patient authentication at log-on to the service (PIN, 4 digits, input using the remote control)

– Timeout: Automatic logoff of user after a given period of idleness

– Encrypted storage of sensitive user data in the Residential Patient Device (RPD)

– Encrypted transfer of messages

– Encrypted VPN for video conferencing

– The RPD was configured as a dedicated computer permitting network access only via selected ports and protocols. The device could only be reached from outside through a VPN or SSH port.

– Health diary information is stored both locally and remotely in order to secure availability

– Education of users, including awareness raising, information about privacy risks, and education in secure behaviour

### Limitations

There are always questions about *completeness* when conducting a risk assessment: Did we identify all risks? Did we find the most important risks? The answer to the first question is probably “no”, but we consider the answer to the second question to be “yes”. The systematic, structured process for threat identification, together with thorough documentation, helps to ensure consistency and improve completeness. The most important effect of the risk assessment is perhaps the awareness it imposes on the designers and developers – and on the users, of possible risks to their system.

The concern about completeness may also be related to performing a qualitative risk assessment. Our approach is qualitative, which we find more suitable for the types of services and information systems we assess. However, one drawback of qualitative risk analysis methods is that their nature yields inconsistent results. The results may be subjective when using qualitative risk analysis methods [20], and there is uncertainty associated with this subjective judgment [22].

A question that may be asked regarding the subjective judgment in our risk assessment is: Were we too strict in the evaluation of risks? For instance, it may be debated whether threats with severe consequence but low likelihood (see risk matrix in Figure 1) should be defined as moderate or low risk. In our case we concluded that this is *moderate* risk, because of the severe consequence.

### Generalisation

Another relevant question concerning the risk assessment is related to *generality*: Can the result be reused for other technical solutions or similar services?

The validation of impact of confidentiality threats relative to threats to other information security aspects may vary between countries. The privacy legislation in most of the European countries is based on the European privacy directive (95/46/EC, [10]), but it is a matter of the national authorities to implement it in their own legislation and manage the compliance of the regulations. In Norway, strong emphasis is put on privacy and confidentiality, also in the healthcare sector.

Within healthcare policy, there is an ongoing discussion about how to achieve a shift from centre-based rehabilitation to rehabilitation at home with the involvement of healthcare workers; and further to self-managed rehabilitation, training, and exercise; reducing the involvement of healthcare staff [1,2]. – This risk assessment had a focus on risks in the home environment, and many of the identified threats will apply to home-based solutions in general. The risks to confidentiality are different at home than in a more controlled (closed) environment: on the one hand, one is more private indoors at home; on the other hand it is the persons in the immediate entourage who can most easily obtain sensitive information about the patient. The patient may want to hide sensitive information from close relatives, yet these may be the very people who are best able to support patients with chronic conditions.

On the technical side, equipment located in private homes is more exposed to access by unauthorised persons. For instance, other persons in the same room, whether they are family members or casual visitors, may obtain sensitive information from this service. The equipment is physically available and thus more likely to be used (or tampered with) by other people in the family. Enabling electronic communication between equipment in the patients’ homes, connected to the open Internet, and equipment placed in the secure zone of a network (e.g. at a hospital), is another security challenge.

Until now, the capabilities of commercial TV set-top-boxes (STB) have been somewhat limited and usually tailored to TV functionality, such as program guides, simple web browsing, and recording of broadcast TV to hard disk. However, the next generation of TVs or STBs will support Hybrid Broadcast Broadband TV or “HbbTV”. The latest innovations include smart TV technologies where the TV has its own broadband Internet connection and supports applications (apps) of the type we know from the mobile world (Apple TV, Google TV, Samsung smart TV). These TV-apps provide rich functionality on the TV, with content and functions from the back-end Internet. These smart TVs do not have touch-sensitive screens, so user interactions are controlled by means of the TV’s remote control. Most of the threats identified in this risk assessment will apply to STBs and smart TVs, as well.

Many of the threats also apply to solutions and services intended for mobile devices. There are, however, other typical threats when dealing with mobile equipment; these devices are easily mislaid, lost or stolen, thus creating extra risks, especially to confidentiality and availability.

The use of home technology and mobile solutions will increasingly supplement and to some extent replace the physical encounter between patient and healthcare professionals. With proper use, this can enhance the privacy of individuals, but it also provides new challenges. Some of the responsibility must remain with the users. The patient must be made aware about potential risks, and the way in which new technology used in home healthcare might affect the individual’s privacy [30].

The increased use of personal and mobile electronic devices inevitably leads to changing attitudes among the population, regarding privacy – ranging from higher awareness on one extreme, to greater carelessness on the other extreme. Similarly, attitudes concerning what are regarded as private or sensitive information may also be changing. As well as being a question of legislation, this is an individual/subjective question. Some users may want information to remain private even if it is not legally defined as sensitive information. For example, a woman who has been badly injured by a violent husband will regard her new address as more “sensitive” than information about her broken arm. On the other hand, many users will say “I have nothing to hide”, and some patients do already present their whole case history on the open Internet (Facebook, YouTube, Patients-like-me, etc.) [31-33].

## Conclusion

A total of approximately 50 threats and potential unwanted incidents were identified and analysed in the risk assessment. The *confidentiality* threats were evaluated as being the most serious threats. This reflects the fact that health-related personal information is regarded as sensitive. All the *availability* threats were classified as having a low risk. That is because the aim of the home programmes is to provide education and rehabilitation services; it is not intended for use in acute situations or for continuous health monitoring.

Only one threat was ascribed an unacceptable *high* risk level. It concerns the case that a third party, e.g. a visitor in the patient’s home, might by chance access personal health information on the TV screen if the system was left switched on and logged in. A solution to this threat is to implement a timeout mechanism to be activated after a specified period without interaction between user and system. The rest of the threats were identified as *medium* or *low* risk.

Most of the threats identified are representative of healthcare services intended for patients or citizens in their homes. The risks to confidentiality are different at home than in a more controlled environment such as a hospital, and electronic equipment situated in private homes and communicating via the open Internet is more exposed to access by unauthorised persons.

By implementing the proposed measures, it has been possible to design a home-based service which ensures the necessary level of information security and privacy.

## Abbreviations

COPD: Chronic obstructive pulmonary disease; DoS: Denial of service; HIPAA: Health insurance portability and accountability act; HW: Hardware; ID: Identifier; ISO: International Standards Organisation; PIN: Personal identification number; PKI: Public key infrastructure; QoS: Quality of service; RPD: Residential patient device; STB: Set-top-box; SW: Software; VC: Video conference; VPN: Virtual private network.

## Competing interests

There are no financial or personal relationships with people or organisations that inappropriately influence this work as far as we know.

## Authors’ contribution

TMB, LVK, and EJ all participated in the risk assessment. EH was the risk assessment leader. EH, TMB, LKV, and EJ all took part in preparing the manuscript, and all authors have approved the final version.

## Pre-publication history

The pre-publication history for this paper can be accessed here:

http://www.biomedcentral.com/1472-6947/13/85/prepub
